# Sustainable Use of Natural Resources and Traditional Medicine in Tropical Countries: Uncovering the Main Antioxidant Compounds and Antihypertensive Potential of the *Diospyros comorensis* Leaves as Health-Promoting Food Application for Local Population

**DOI:** 10.3390/plants15111757

**Published:** 2026-06-05

**Authors:** Ahmed Ali, Dario Donno, Zoarilala Rinah Razafindrakoto, Nantenaina Tombozara, Azali Ahamada-Himidi, Mamy Julien Randrianirina, Giovanni Gamba, Jean François Rajaonarison, Gabriele Loris Beccaro, David Ramanitrahasimbola

**Affiliations:** 1 Laboratoire Aliments, Réactivité et Synthèse des Substances Naturelles, Faculté des Sciences et Techniques, Université des Comores, Moroni 167, Comoros; liahameda@gmail.com (A.A.); azali_a@yahoo.fr (A.A.-H.); 2 Ecole Doctorale, Génie du Vivant et Modélisation, Université de Mahajanga, Mahajanga 401, Madagascar; jef.rajaonarison@gmail.com; 3 Dipartimento di Scienze Agrarie, Forestali e Alimentari, Università Degli Studi di Torino, 10095 Grugliasco, TO, Italygabriele.beccaro@unito.it (G.L.B.); 4 Institut Malgache de Recherches Appliquées, Antananarivo 101, Madagascar; zo_ari_lala@yahoo.fr (Z.R.R.); nzara89@gmail.com (N.T.); mjrandrianirina@gmail.com (M.J.R.); ramanitrahasimboladavid@gmail.com (D.R.); 5 Pharmacy Department, Faculty of Medicine, University of Antananarivo, Antananarivo 101, Madagascar

**Keywords:** *Diospyros comorensis*, cardiovascular effects, phenolic compounds, ellagic acid, quercetin, antioxidant activity, diuretic activity, vasorelaxant activity, natural medicine

## Abstract

*Diospyros comorensis* Hiern is a medicinal plant traditionally utilized in the management of cardiovascular disorders. Despite its common use, the pharmacological properties and phytochemical composition remain unexplored. This study aimed to evaluate the vasorelaxant, diuretic, and antioxidant activities, as well as toxicity and phytochemical profiling, of a methanol–water extract of *D. comorensis* leaves (MDCR) and a decoction of *D. comorensis* leaf (DDCR) extract. The main phytochemicals were quantified using High-Performance Liquid Chromatography (HPLC). Antioxidant capacity was assessed using DPPH and FRAP assays. The vasorelaxant effect was evaluated in vitro on phenylephrine-precontracted aortic rings. Diuretic activity was determined by measuring Wistar rats’ urine output and electrolyte levels (Na^+^, Cl^−^, and K^+^). Toxicity was assessed using Swiss mice. The extracts showed a total phenolic content (TPC) of 29,693.02 ± 3493.75 mg GAE/100 g DW (Folin–Ciocalteu method), which was markedly higher than the total phenolics quantified by HPLC (3743.12 ± 457.32 mg/100 g DW, representing 76.38% of the total bioactive fraction). Among the quantified constituents, ellagic acid (56.36%) was the main compound. Both extracts exhibited marked antioxidant capacity along with significant vasorelaxant effects on phenylephrine-precontracted rat aorta rings, with EC50 values of 3.83 ± 0.81 µg/mL for MDCR and 4.87 ± 0.79 µg/mL for DDCR. Acute toxicity was not observed with either extract. The identified compounds may be involved in the observed antioxidant and pharmacological effects. These results show experimental evidence useful to support the traditional use of *D. comorensis* leaves in managing high blood pressure and highlight the antihypertensive potential of this Comorian endemic species. Further studies are necessary to characterize the biological mechanisms involved and relative bioactive substances. Reporting the pharmacological activities of *D. comorensis* may contribute to the sustainable use of natural resources in the Comoros Islands and Madagascar.

## 1. Introduction

Arterial hypertension (AHT) remains a primary risk of global cardiovascular morbidity, characterized by persistently elevated blood pressure, with systolic blood pressure (SBP) greater than 140 mmHg and/or diastolic blood pressure (DBP) greater than 90 mmHg [1]. In Africa, the adult prevalence rate varies from 20% to 40% and reflects the growing impact of urbanization and the shift to sedentary lifestyles, alongside specific genetic factors [2,3]. Hypertension acts as a critical determinant of cardiovascular complications, including heart failure, and peripheral vascular diseases, as well as stroke and coronary artery disease [4,5]. Left ventricular hypertrophy and progressive ventricular dysfunction are direct consequences of sustained pressure overload [6]. Therefore, effective management is essential to reduce cardiovascular risks, with current therapies including diuretics, beta-blockers, and vasodilators [7].

Oxidative stress plays a key role in vascular dysfunction and blood pressure dysregulation. For this reason, identifying natural antioxidants represents an excellent strategy for cardiovascular protection. *Diospyros comorensis* is traditionally used in the Comoros for the management of cardiovascular disorders, including hypertension, but its antioxidant and vascular effects have not yet been documented. This ethnomedical background justified the investigation of its leaves as a potential source of bioactive molecules [8].

Medicinal plants remain widely used in developing countries despite the availability of antihypertensive drugs, due to accessibility, perceived safety, cultural acceptance, and low cost. Moreover, the sustainable use of plant resources in traditional folk medicine represents an important strategy to preserve biodiversity and support local healthcare systems in tropical islands such as Madagascar and the Comoros [9,10]. *Diospyros comorensis* Hiern, an endemic species of the Comoros Islands, belongs to the Ebenaceae family. It is traditionally used to treat heart disorders, hypertension, and inflammatory symptoms [11]. However, comprehensive phytochemical and pharmacological data on this species remain limited, while several studies reported the chemical composition and relative biological properties of other Diospyros species, such as D. kaki and D. lotus. These species contain phenolics, flavonoids, and triterpenoids associated with antioxidant and therapeutic effects [12,13].

The aim of this study was to evaluate the antioxidant capacity, cardiovascular activity, and acute toxicity of *D. comorensis* leaves, as well as to identify their main bioactive compounds, in order to provide scientific evidence for supporting their traditional use. This work may also contribute to the valorization of *D. comorensis* as a potential source of natural health-promoting agents targeting hypertension and oxidative stress-related diseases, also supporting its ethnopharmacological relevance and the need for sustainable utilization of this species.

## 2. Results and Discussion

### 2.1. Extraction

Methanol–water maceration produced the MDCR extract with a yield of 18.27 ± 0.68%, while the aqueous decoction produced the DDCR extract with a yield of 25.20 ± 0.42% (yields were obtained from three extraction batches for each extraction method, *N* = 3). In this study, the higher yield obtained with DDCR suggests that hot water extraction was more efficient in recovering water-soluble constituents from *D. comorensis* leaves, including polysaccharides and other polar compounds. Hot water extraction is very efficient for compounds with high molecular weight due to the extended time of thermal action, which promotes the breakdown of plant cell walls and facilitates the solubilization of specific compounds, such as polysaccharides; the extraction of these compounds is more difficult at lower temperatures by cold maceration due to a reduced solvent penetration, and a limited diffusion reduces molecule release [14,15]. In contrast, the methanol–water mixture may allow the recovery of a broader range of phenolic and thermolabile compounds. The combination of water with water-miscible organic solvents, such as methanol or ethanol, even if less effective for the extraction of some high-molecular-weight substances, may preserve thermolabile components and optimize the extraction of substances soluble in the mixture, allowing the recovery of a larger range of chemical compounds in the resulting extracts, such as phenolics, vitamin C, and organic acids [16]. Moreover, Rhazi et al. [17] reported that excessively long extraction times may degrade some natural molecules and substances, such as polyphenols and vitamin C, thereby affecting the overall yield and quality. Therefore, both extracts were considered in the study to compare the traditional aqueous preparation with a hydroalcoholic extract suitable for phytochemical characterization.

### 2.2. Total Phenolic Content (TPC)

The TPC of *D. comorensis* leaves was quantified as 29,693 ± 3493.75 mg GAE/100 g DW, highlighting significant amounts of phenolics in the considered plant material. Phenolic compounds represent the most abundant polar class of secondary metabolites in plants, and their extraction is highly influenced by selected extraction conditions and solvent polarity. In this study, their high recovery was consistent with the efficiency of the used extraction procedures. The reported TPC values also highlighted significant levels of bioactive molecules, such as polyphenols, with high health-promoting and antioxidant potential in *D. comorensis* leaves. Moreover, the high standard deviation may be due to the high natural variability of phenolics during their accumulation in plants, due to plant maturity and/or environmental factors, together with the effects based on leaf processing and handling [18,19]. This value is notably higher than those reported for other plant species using the same protocol, including *Imperata cylindra* (1920.63 ± 360.62 mg GAE/100 g DW) and *Lygodium lanceolatum* (853.68 ± 203.89 mg GAE/100 g DW), highlighting that *D. comorensis* leaves may be important potential sources of phenolic compounds [20,21]. For this reason, this species may be regarded as a promising candidate for future pharmacological studies and nutraceutical investigations focused on the production of innovative foods, health-promoting beverages, and herbal applications.

### 2.3. Fingerprints Compounds in the Leaves of D. comorensis

Among the 24 bioactive compounds selected, eight were quantified in the leaves of *D. comorensis*, and their amounts were expressed as milligrams per 100 g DW (Table 1). The sum of all the quantified compounds (4900.65 ± 315.32 mg/100 g DW) was considered the total bioactive compound content (TBCC). This value indicated a significant presence of health-related phytochemicals in the plant. The calculated quantified phenolic content (i.e., the sum of phenolic acids, catechins, and flavonols) was 3743.12 ± 457.32 mg/100 g DW, accounting for 76.38% of TBCC, and demonstrated that phenolic compounds constitute the main chemical class in *D. comorensis* leaves. This value, lower than the TPC values (29693 ± 3493.75 mg GAE/100 g DW), suggested that this species contains additional phenolic compounds not included in the list of biomarkers in Table 1 [18,22]. Moreover, the Folin–Ciocalteu method is an aspecific method, and many molecules, not only phenolics, may contribute to the final amounts, overestimating the real phenolic levels if compared to HPLC results [21]. This difference between targeted compound quantification and TPC determination has also been reported in other plant matrices [20,22]. The bioactive compound profile of *D. comorensis* leaves, expressed relative to TBCC, is presented in Table 1. Ellagic acid was the predominant compound (56.39% of TBCC), followed by quinic acid (23.57%) and quercetin (11.22%), then epicatechin (4.96%) and catechin (3.55%). Gallic acid (0.25%), fructose (0.03%), and glucose (0.02%) were detected in trace amounts. The dominance of polar phenolics such as ellagic acid and quercetin is consistent with other studies on plant foods and by-products, where similar phenolic profiles have been associated with strong antioxidant capacity and potential health benefits [23].

### 2.4. Antioxidant Capacity

MDCR and DDCR presented a strong free radical DPPH scavenging capacity with respective IC_50_ values of 49.67 ± 4.44 and 28.69 ± 3.14 µg/mL; gallic acid, used as a positive control, showed a value of 15.73 ± 0.23 µg/mL. The higher DDCR antioxidant power may be due to the significant affinity of polar compounds, mainly phenolics, for water. Indeed, these molecules substantially contribute to the antioxidant capacity of different plant extracts [24,25]. Similar results have been reported for several Lamiaceae species, including *Mentha pulegium*, *Origanum vulgare*, and *Thymus* spp. (e.g., *T. vulgaris* and *T. serpyllum*) [26]. The antioxidant capacity of MDCR and DDCR was markedly stronger when compared with those of several plant extracts tested using the same method, such as the methanolic extracts of *Schefflera bojeri* (bark and leaves), which showed IC_50_ values of 480.71 ± 1.46 and 184.76 ± 1.68 µg/mL, respectively [27], or the methanolic extract of *V. secundiflorum* (aerial parts) that presented a value of 76.06 ± 1.08 µg/mL [18]. The antioxidant capacity of the *D. comorensis* leaf extracts was further confirmed by the FRAP assay, which yielded a value of 1750.69 ± 21.13 mmol Fe^2+^/kg DW. This value was notably higher when compared to those obtained for other species, including *S. bojeri* leaves and bark (68.57 ± 0.41 and 57.54 ± 2.40 mmol Fe^2+^/kg DW), *V. secundiflorum* aerial parts (69.31 ± 3.31 mmol Fe^2+^/kg DW), and *Morella spathulata* leaves and bark, with respective values of 227.89 ± 12.53 and 376.18 ± 115.69 mmol Fe^2+^/kg DW [18,23]. Most of the bioactive substances and compounds identified by HPLC analysis consisted of organic acids and phenolics, which are recognized for their antioxidant capacity—in particular, ellagic acid, quercetin, and catechins [28,29,30].

These findings suggested that the strong antioxidant potential of *D. comorensis* leaves may be closely linked to their high phenolic content. These preliminary results may be a significant base for further studies to evaluate the traditional use of this plant and its leaf-derived herbal beverages in managing oxidative stress-related cardiovascular conditions.

### 2.5. Diuretic Effects of MDCR and DDCR

**Figure 1:**
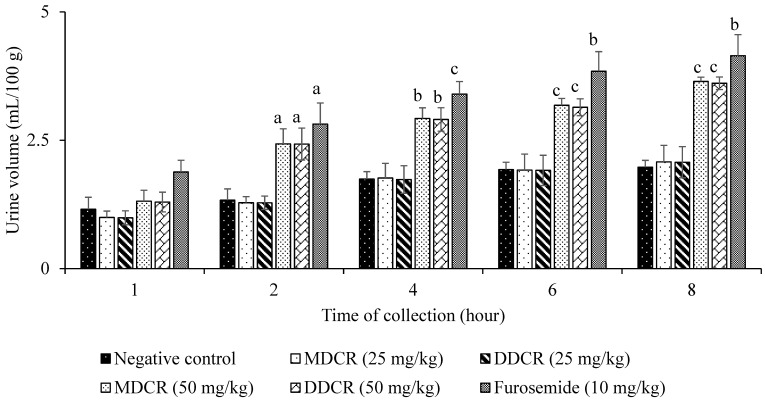
Cumulative urinary volume of animals treated with MDCR, DDCR, or furosemide compared to the negative control group. (Values are represented by mean ± SEM of 5 independent determinations; a: *p* < 0.05; b: *p* < 0.01; c: *p* < 0.001).

In this case, as well as in the other pharmacological tests, both MDCR and DDCR were tested to compare the effective power of the herbal beverage (aqueous decoction) used by the local population, compared to the herbal preparation resulting from the best extraction method (methanolic extract). The diuretic effects of MDCR (25 and 50 mg/kg) and furosemide (10 mg/kg) are highlighted in Figure 1. Treatment with furosemide significantly increased the cumulative urine volume at the different collection times compared with the negative control (*p* < 0.05), except at 1 h, where the increase was not significant (*p* > 0.05), thereby confirming the effectiveness of the protocol. The effects of DDCR (25 and 50 mg/kg) were also evaluated under the same experimental conditions. Treatment with MDCR at 25 mg/kg did not significantly affect urine volume at any collection time, while MDCR at 50 mg/kg significantly increased urine volume from the second to the eighth hour of collection (*p* < 0.05), showing the diuretic effects of *D. comorensis* leaf extract. Similarly, DDCR at 25 mg/kg did not significantly modify urine volume, whereas DDCR at 50 mg/kg significantly increased urine output from the second to the eighth hour (*p* < 0.05). No significant difference was observed between MDCR and DDCR at equivalent doses (*p* > 0.05), indicating comparable diuretic efficacy of both extracts. DCR extract diuresis at a higher dose (50 mg/kg) might be due to the presence of different phenolic compounds quantified in the extracts, such as catechin, quercetin, and gallic acid, even at relatively lower concentrations [31,32]. The comparable activity observed with DDCR further supports the role of these water-extractable phenolic constituents in mediating the diuretic effect. Similar studies have shown that the efficacy of diuretics is often dose-dependent, requiring sufficient plasma concentrations to elicit a significant physiological response [33]. MDCR diuretic effects at the dose of 50 mg/kg were comparable to those of furosemide at the dose of 10 mg/kg at each urine collection time (*p* > 0.05; Figure 1). A similar trend was observed for DDCR at 50 mg/kg, which also showed comparable effects to furosemide (*p* > 0.05). Furosemide is a reference loop diuretic that inhibits the reabsorption of chloride, potassium, and sodium ions in the loop of Henle, thereby increasing urinary excretion of water and sodium, which leads to blood volume and blood pressure reduction [34]. To further investigate this mechanism, the electrolyte concentrations (Na^+^, K^+^, and Cl^–^), as well as the pH of the collected urine after 8 h of treatment, were measured. The results are presented in Table 2.

A non-significant increase in sodium, potassium, and chloride ion concentrations and pH was observed for MDCR or DDCR treatment at 25 mg/kg (*p* > 0.05), while significant increases in sodium, potassium, and chloride ion concentrations and pH were observed for 50 mg/kg and furosemide treatment (*p* < 0.05, respectively) when compared to the negative control group. Therefore, all DCR extracts demonstrated significant natriuretic, kaliuretic, and chloruretic effects. When compared to that of furosemide, MDCR or DDCR (50 mg/kg) diuresis was similar, allowing the study to postulate that they may show the same diuretic mechanism. However, the mechanism of blocking the Na^+^/Cl^–^ cotransporter, similarly to that of diuretic thiazides, cannot be ruled out. Furosemide, a loop diuretic, specifically targets the Cl^−^/ K^+^/Na^+^ cotransporter, reducing the reabsorption of chloride (Cl^−^), potassium (K^+^), and sodium (Na^+^) in the kidneys, which leads to a significant increase in their urinary excretion [34]. However, further studies on the interaction of metabolites contained in DCR with these ion channels would be necessary to confirm this preliminary result. MDCR is rich in phenolics and organic acids, which may be responsible for these observed effects. Gallic acid, quercetin, and other flavonoids have been reported to downregulate the epithelial Na^+^ channel (ENaC) renal expression, playing a central role in Na^+^ reabsorption within the renal tubules. In addition, quercetin has been shown to modulate the activity of Na^+^/K^+^/2Cl^−^ cotransporter 1 (NKCC1), an important regulator of cytosolic Cl^−^ concentration, thereby facilitating diuresis and natriuresis, both crucial processes in their antihypertensive effects [35,36]. By helping to prevent or alleviate renal damage associated with hypertension, these compounds act by directly targeting renal tissue and reducing blood pressure. Furthermore, phenolic compounds are very important in the diuretic effect observed in some plant species, particularly by inducing vasodilation of the afferent arterioles in the renal vasculature [36]. This vasodilation leads to an increase in the glomerular filtration rate, thus promoting increased urine production. Additionally, these compounds also inhibit carbonic anhydrase enzyme activity in the renal tubule [37], further contributing to increased diuresis. Flavonoids and phenolic compounds also act by inhibiting the angiotensin-converting enzyme (ACE), which enhances urine production [38]. These compounds improve the bioavailability of bradykinin, prostacyclin, and nitric oxide, or alternatively, exert an inhibitory effect on Na^+^/K^+^-ATPase activity, contributing to the modulation of renal excretion.

On the other hand, a slight alkalinization of the pH was noted in the urine of the MDCR- or DDCR (50 mg/kg)-treated animals when compared to those in the negative control group (*p* < 0.05), which was not the case in furosemide-treated animals. Urinary pH plays an important role in proper renal function. The kidneys regulate blood acid/base balance by excreting protons into the urine. An overly acidic or alkaline urine pH can disrupt this balance, affect body homeostasis, and modify several physiological functions [39]. Additionally, urine pH may influence the excretion of specific drugs and metabolites by ionization, thus accelerating their elimination and modifying their efficacy. For instance, urine alkalinization may increase the excretion of weak acids such as acetylsalicylic acid [40]. In specific studies on plant diuretic activity, urinary pH analysis is particularly important, as it reflects the body acid–base balance and may affect the effectiveness of diuretics [41].

### 2.6. Vasorelaxant Effects of D. comorensis Extracts

**Figure 2:**
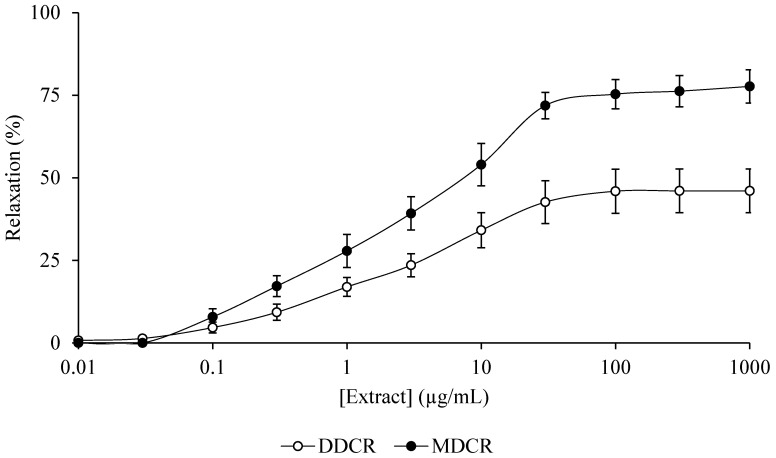
Concentration-effect curves of MDCR and DDCR on PE pre-contracted isolated rat aorta (n = 6).

MDCR and DDCR exerted concentration-dependent relaxation on PE pre-contracted rat aorta, as shown in Figure 2, indicating that *D. comorensis* leaves contain bioactive compounds responsible for their vasorelaxant activity. Their EC_50_ and maximal effect (E_max_) values are reported in Table 3. MDCR exhibited a lower EC_50_ value (3.83 ± 0.81 µg/mL) than DDCR (4.87 ± 0.79 µg/mL) with no statistical differences (*p* > 0.05). In any case, the E_max_ of MDCR (77.67 ± 5.03%) was significantly higher than that of DDCR (45.89 ± 6.11%, *p* < 0.01). These results suggest similar potency (EC_50_) but different maximal efficacy (E_max_), indicating a quantitative rather than qualitative difference in vasorelaxant activity. This may reflect differences in the concentration of bioactive constituents between MDCR and DDCR. These findings suggested that compounds in *D. comorensis* leaves may contribute to its antihypertensive effects by reducing peripheral arterial resistance and lowering blood pressure. Such effects may be attributed to some phenolic constituents, including ellagic acid, gallic acid, quercetin, catechins, and other related compounds [42,43,44,45,46]. Vascular smooth muscle cell (VSMC) relaxation might occur through the direct activation of receptors on the smooth muscle cell membrane or might occur indirectly via activation of endothelial cell receptors. The indirect pathway involves endothelial-released nitric oxide (NO), which subsequently activates soluble guanylate cyclase in the VSMCs, resulting in vasorelaxation [47]. In both cases, vasorelaxation could be involved in intracellular signaling pathways mediated by cyclic adenosine monophosphate (cAMP) or cyclic guanosine monophosphate (cGMP), which activate cAMP-dependent protein kinase (PKA) and cGMP-dependent protein kinase (PKG), respectively. The outcome of these signaling cascades may lead to the dephosphorylation of the myosin light chain by the myosin light chain phosphatase, thereby facilitating smooth muscle relaxation [48]. In the present study, although an endothelium-dependent component of relaxation may be suggested based on the observed responses, the involvement of specific pathways, such as NO synthesis, cyclooxygenase-derived prostanoids, or potassium channel activation, was not experimentally investigated. Therefore, the contribution of these signaling mechanisms remains to be elucidated and should be considered as a working hypothesis requiring further confirmation by using selective pharmacological inhibitors (e.g., L-NAME, indomethacin, or K^+^ channel blockers). The presence of ellagic acid and quercetin in *D. comorensis* leaves further supports their ability to induce endothelium-dependent vasorelaxation [43,44]. Moreover, the antioxidant properties of *D. comorensis* leaves may play a significant role in hypertension management, a condition characterized by increased peripheral vascular resistance resulting from elevated vascular tone or structural alterations in resistance arteries, partly through the regulation of NO bioavailability [49,50].

### 2.7. Acute Toxicity of DCR

The acute toxicity treatment with MDCR and DDCR, ranging from 500 to 2000 mg/kg, did not show any toxicity effects relative to the observed parameters, including skin coloration, hair erection, respiratory system, defecation functions, food intake, or other behaviors in treated mice. No behavioral abnormalities or mortality were reported during the 48 h observation period for all the groups. Therefore, the median lethal dose (LD_50_) of MDCR and DDCR should be more than 2000 mg/kg, confirming the absence of toxicity of the leaves of *D. comorensis* according to the OECD guideline 423 [51]. This observation may confirm the safety of the use of this species, as claimed by Comorian traditional practitioners and common people. Additionally, several species used by other traditional practitioners from different countries have been demonstrated to be non-toxic by testing their extracts at higher concentrations than those recommended by the OECD [18,20,27,52].

## 3. Materials and Methods

### 3.1. Plant Materials and Sampling

*D. comorensis* leaves were collected in April 2022 at Hahaya (11°33′55″ S; 43°16′35″ E, 50.24 m above sea level) in the Itsandra Hamavou region (Ngazidja island, Comoros). The voucher specimen was identified by Dr Mohamed ANDILIYAT, a botanist at the University of Comoros and the National Herbarium of Comoros, by a comparison with a reference sample (AND301) preserved in the National Herbarium of the Comoros and deposited at the National Herbarium (n° DCR001/2022). Collected leaves were dried for two weeks at room temperature in a dry and aerated place to avoid moisture before being ground into a fine powder.

### 3.2. Animals

Male Wistar rats (3–4 months old; 150–200 g) and Swiss mice (4–6 weeks old; 23–27 g) were used to evaluate vascular activity and toxicity, respectively. The animals were housed under controlled conditions (12 h dark/light cycle, 25 ± 2 °C temperature, and 50 ± 10% humidity) at the animal facility of the *Institut Malgache de Recherche Appliquée* (IMRA). They were fed with a standard food pellet (1420, Livestock Feed Ltd., Port Louis, Mauritius) and had *ad libitum* access to water. Before the experimentation, animals were fasted overnight. All the experimental procedures were carried out following the European Directive on the safety and protection of animals used for scientific studies (DIRECTIVE 2010/63/EU), and they were approved by the local ethics committee (n° 003/CEA/IMRA/2022).

### 3.3. Extract Preparation

#### 3.3.1. Maceration

*D. comorensis* leaf powder (100 g) was macerated in 500 mL of a 95:5 (*v*/*v*) methanol–water mixture for 24 h with continuous agitation. The mixture was then filtered. The filtrate was recovered, and the marc was again macerated using the same procedure. Finally, filtrates were gathered and evaporated under reduced pressure to dryness using a rotary evaporator (Büchi R-114, Büchi Labortechnik AG, Flawil, Switzerland) at 40 °C to obtain the methanol extract of the leaves of *D. comorensis* (MDCR).

#### 3.3.2. Decoction

Another amount of *D. comorensis* leaf powder (20 g) was suspended in distilled water (1 L). The mixture was then boiled for 10 min and filtered. Filtrate was evaporated to dryness using the previous rotary evaporator at 40 °C under reduced pressure to obtain the decoction of the leaves of *D. comorensis* (DDCR).

The dried extracts were stored in airtight containers, protected from light and humidity, and kept at 4 °C temperature until further experimental use. The dried extracts were reconstituted in distilled water or in an appropriate vehicle according to the requirements of each biological assay.

### 3.4. Chromatographic Analysis

Phytochemical composition of the extracts was evaluated by chromatographic analysis. Chromatographic separation was performed using an Agilent 1200 High Performance Liquid Chromatography (HPLC) system (Agilent Technologies, Santa Clara, CA, USA) coupled to a UV/Vis diode array detector. Twenty-four bioactive compounds were selected to be identified in the plant materials for their relevance to human health [53]. More detailed information on the chromatographic analysis is reported in the Supplementary Materials. Calibration data, method validation information, and more detailed notes on chromatographic conditions are reported in the Supplementary Materials (Tables S1 and S2).

### 3.5. Quantification of the Total Phenolic Content (TPC)

The Folin–Ciocalteu method [54] was used for total polyphenolic content (TPC). Absorbance was evaluated at 760 nm, and the results were expressed as mg gallic acid equivalents (GAE) per 100 g of dry weight (DW). Gallic acid calibration solutions were prepared in methanol at concentrations ranging from 0.02 to 0.10 mg/mL [53].

### 3.6. Antioxidant Capacity Evaluation

MDCR and DDCR antioxidant potential was assessed using both the 2,2-diphenyl-1-picrylhydrazyl (DPPH) assay described by Sreejayan and Rao [55] with slight modifications and the Ferric Reducing Antioxidant Power (FRAP) assay described by Tombozara et al. [18]. More detailed information is reported in the Supplementary Materials.

### 3.7. Diuretic Assay

To evaluate the diuretic effects of MDCR and DDCR, the protocol described by Sanou et al. [56] was slightly modified. Four groups of five fasted rats were orally treated with 25 mL/kg saline solution (0.9% NaCl). The following treatments were orally administered in a volume of 10 mL/kg, prepared in saline solution immediately after saline solution administration:

- Group I: the control that received saline solution;
- Group II: the positive control that received furosemide at a dose of 10 mg/kg;
- Groups III and IV: the groups treated with MDCR at the doses of 25 and 50 mg/kg, respectively.
- Groups V and VI: the groups treated with DDCR at the doses of 25 and 50 mg/kg, respectively.

Their urine was collected using a test tube at 1, 2, 4, 6, and 8 h after the administration of the test sample. The cumulative urine volume was measured at these different times and expressed as mL/100 g of body weight (bw). The electrolyte contents, including Na^+^, K^+^, and Cl^−^, were evaluated by an atomic absorption spectrophotometer (iCE 3300 AAS, ThermoFisher, Waltham, MA, USA), and their pH was assessed at the end of the experiment by a pH meter (Mettler Toledo, Columbus, OH, USA).

### 3.8. Vascular Assay

MDCR and DDCR effects on vascular smooth muscle were assessed on phenylephrine (PE) pre-contracted rat aorta using the method described by Rakotodramanana et al. [57].

#### 3.8.1. Aorta Preparation

The rat was euthanized with ether, then exsanguinated. Its thoracic aorta was then isolated and placed into a Petri dish containing Krebs–Henseleit solution (NaCl 118.0 mM, KCl 4.7 mM, NaHCO_3_ 25.0 mM, CaCl_2_ 1.8 mM, NaH_2_PO_4_ 1.2 mM, MgSO_4_ 1.2 mM, D-glucose 11.0 mM, and H_2_O), which was continuously aerated with carbogen (95% O_2_, 5% CO_2_). After removing the adipose tissue, the aorta was sectioned into 2–3 mm rings. These segments were placed in the organ bath with the Krebs solution, maintained at a temperature of 37 °C and continuously aerated with an O_2_/CO_2_ mixture. Each aortic ring was then tensioned under 1 g, allowing it to equilibrate for 90 min; during this period, the survival solution was renewed every 30 min. The rings were subsequently pre-contracted with 10^−5^ M of phenylephrine (PE) and relaxed with acetylcholine (10^−6^ M). Finally, the tension was adjusted to 2 g to assess the contractility of the rings.

#### 3.8.2. Vasorelaxant Activity Assessment

Rings were contracted with PE (10^−6^ M). When the contraction plateau was reached, increasing and cumulative concentrations varying from 10^−5^ to 1 mg/mL of MDCR or DDCR were added in the organ bath with an interval of 4 min. The variation of contractile forces of the aortic rings was measured using a BUXCO tension amplifier–recorder. The effect of each concentration was evaluated as a percentage of relaxation relative to the complete relaxation caused by the contractile agonist, considered the maximal effect. The EC_50_ (concentration producing 50% of the maximal effect) was calculated by linear regression.

### 3.9. Acute Toxicity Study

The toxicity study of MDCR and DDCR was performed on mice. Animals were fasted and then divided into 7 groups consisting of 5 individuals. Group I was orally treated with distilled water (10 mL/kg b.w) and served as a control. Groups from II to IV were treated with MDCR at the respective doses of 500, 1000, and 2000 mg/kg, while groups from V to VII were treated with DDCR at the respective doses of 500, 1000, and 2000 mg/kg in a volume of 10 mL/kg b.w per mouse administered by gavage. After treatment, animals had free access to water and food. The animal behaviors were observed continuously during the first 6 h and then every 12 h for 2 days after the treatment. Any mortality and abnormal behaviors compared to the control animals were noted.

### 3.10. Statistical Analysis

Mean ± standard deviation (SD) or standard error of mean (SEM) was used to express the r, based on n experimental replicates or the tested animal number. Student’s *t*-test and one-way analysis of variance (ANOVA), followed by Tukey’s HSD test for multiple comparisons, were utilized for the data statistical elaboration using SPSS v. 27 software. Differences were considered statistically significant with a *p* < 0.05.

## 4. Conclusions

This study investigated the antioxidant and cardiovascular effects of *D. comorensis* leaves, a plant traditionally used by the Comorian population to treat hypertension, heart failure, and other inflammatory diseases. The leaf extracts exhibited antioxidant, diuretic, and vasorelaxant properties, which may be associated with their phenolic composition (i.e., ellagic acid, quercetin, catechin, and epicatechin). The observed antioxidant and pharmacological properties, together with a favorable safety profile, highlighted the potential of *D. comoriensis* and its leaf-derived herbal beverages as an interesting candidate for development of natural antihypertensive drugs or health-promoting food applications for the local population. Given the reliance on traditional medicine in Comoros and other African countries, these results may identify an innovative natural therapeutic resource, previously undocumented, offering more than an ethnopharmacological validation. Further efforts will focus on bio-guided isolation, structural characterization (NMR, LC-MS/MS), and molecular understanding of the active constituents in *D. comorensis* leaves to confirm these exploratory first observations. Furthermore, mechanistic studies on *D. comorensis*, highlighting endothelium-dependent vs. endothelium-independent, will be considered in the future, as well as a sub-acute toxicity evaluation to further assess safety and support its potential as a therapeutic application or functional food.

## Figures and Tables

**Table 1:** Bioactive compounds quantified by the developed HPLC analysis in the leaves of *D. comorensis*.

Class	Bioactive Marker	Mean Value ^a^	Standard Deviation	Yield (%) ^b^
Cinnamic acids	Caffeic acid	n.d.	/	/
	Chlorogenic acid	n.d.	/	/
	Coumaric acid	n.d.	/	/
	Ferulic acid	n.d.	/	/
Flavonols	Hyperoside	n.d.	/	/
	Isoquercitrin	n.d.	/	/
	Quercetin	549.67	14.64	11.22
	Quercitrin	n.d.	/	/
	Rutin	n.d.	/	/
Benzoic acids	Ellagic acid	2763.86	44.11	56.39
	Gallic acid	12.19	0.49	0.25
Catechins	Catechin	174.06	6.63	3.55
	Epicatechin	243.34	3.22	4.96
Calculated phenolic content		3743.12	457.32	76.38
Organic acids	Citric acid	n.d.	/	/
	Malic acid	n.d.	/	/
	Oxalic acid	n.d.	/	/
	Quinic acid	1155.19	6.83	23.57
	Succinic acid	n.d.	/	/
	Tartaric acid	n.d.	/	/
Vitamins	Ascorbic acid	n.d.	/	/
	Dehydroascorbic acid	n.d.	/	/
Sugars	Fructose	1.20	0.18	0.03
	Glucose	1.13	0.12	0.02
	Sucrose	n.d.	/	/
Total bioactive compound content (TBCC)		4900.65	315.32	100

^a^ Mean value is expressed as mg/100 g DW of three repetitions; ^b^ relative proportion to TBCC; n.d.: not detected.

**Table 2:** Electrolyte concentrations and pH values of MDCR and furosemide-treated rat urine after 8 h.

Group	Negative Control	Furosemide	MDCR		DDCR	
Dose (mg/kg)	0	10	25	50	25	50
Na^+^ (mM)	44.40 ± 2.42	71.00 ± 3.65 ^b^	48.70 ± 1.50	60.40 ± 1.33 ^c^	48.78 ± 1.87	59.00 ±10.91 ^c^
K^+^ (mM)	103.98 ± 0.82	152.46 ± 4.50 ^c^	105.38 ± 1.67	142.82 ± 2.95 ^c^	105.17 ± 1.58	141.4 ± 4.59 ^c^
Cl^−^ (mM)	140.00 ± 0.55	162.40 ± 2.29 ^c^	147.70 ± 3.32	188.40 ± 6.53 ^b^	146.4 ± 3.71	187.2 ± 6.97 ^b^
pH	7.96 ± 0.15	8.01 ± 0.07	8.16 ± 0.04	8.38 ± 0.04 ^a^	8.14 ± 0.06	8.15 ± 0.10 ^a^

Results are expressed as mean ± SEM (n = 5). ^a^: *p* < 0.05 vs. negative control; ^b^: *p* < 0.01 vs. negative control; ^c^: *p* < 0.001 vs. negative control.

**Table 3:** EC50 and E_max_ values of MDCR and DDCR (n = 6).

Tested Extracts	EC_50_ (µg/mL)	E_max_ (%)
MDCR	3.83 ± 0.81	77.67 ± 5.03
DDCR	4.87 ± 0.79	45.89 ± 6.11 ^a^

Values are expressed as mean ± SEM (n = 6). ^a^: *p* < 0.01 vs. MDCR.

## Data Availability

The original contributions presented in this study are included in the article/Supplementary Materials. Further inquiries can be directed to the corresponding author.

## Supplementary Materials

The following supporting information can be downloaded at: https://www.mdpi.com/article/10.3390/plants15111757/s1, Table S1: Calibration curve, R2, LOD, and LOQ of each selected bioactive compound; Table S2: Chromatographic conditions of each used method.

## Abbreviations

The following abbreviations are used in this manuscript:

ACE	Angiotensin-converting enzyme
AHT	Arterial hypertension
ANOVA	Analysis of variance
b.w	Body weight
cAMP	Cyclic adenosine monophosphate
cGMP	Cyclic guanosine monophosphate
DBP	Diastolic blood pressure
DDCR	Aqueous leaf extract of D. comorensis (decoction of the leaves)
DPPH	2,2-diphenyl-1-picrylhydrazyl
DW	Dry weight
EC50	Concentration that produces 50% of the maximal effect
Emax	Maximal effect
ENaC	Epithelial sodium channel
FRAP	Ferric reducing antioxidant power
GAE	Gallic acid equivalents
HPLC	High-performance liquid chromatography
IC50	Concentration demonstrating 50% inhibition
IMRA	Institut Malgache de Recherche Appliquée
LC-MS/MS	Liquid Chromatography–tandem Mass Spectrometry
LD50	Median lethal dose
MDCR	Methanolic leaf extract of D. comorensis
n.d	Not detected
NKCC1	Na^+^/K^+^/2Cl^−^ cotransporter 1
NMR	Nuclear Magnetic Resonance
NO	Nitric oxide
OECD	Organisation for Economic Co-operation and Development
PE	Phenylephrine
pH	Potential of hydrogen
PKA	cAMP-dependent protein kinase
PKG	cGMP-dependent protein kinase
SBP	Systolic Blood Pressure
SEM	Standard error of the mean
TBCC	Total bioactive compound content
TPC	Total phenolic content
TPTZ	2,4,6-tripyridyl-s-triazine
VSMCs	Vascular smooth muscle cells
