# Supplementary material for: Sustainable Use of Natural Resources and Traditional Medicine in Tropical Countries: Uncovering the Main Antioxidant Compounds and Antihypertensive Potential of the Diospyros comorensis Leaves as Health-Promoting Food Application for Local Population
Source: Plants (Basel). 2026 Jun 5;15(11):1757. doi: 10.3390/plants15111757 (PMC13259053; doi:10.3390/plants15111757)
Supplement: Supplementary file 1 [file plants-15-01757-s001.zip › Suppl materials_revised_270526_030626.pdf]

plants

Article

Sustainable Use of Natural Resources and Traditional Medicine in Tropical Countries: Uncovering the Main Antioxidant Compounds and Antihypertensive Potential of the *Diospyros comorensis* leaves as Health-Promoting Food Application for Local Population

Ahmed Ali[1,4], Dario Donno[2,*], Zoarilala Rinah Razafindrakoto[3], Nantenaina Tombozara[3], Azali Ahamada-Himidi[1], Mamy Julien Randrianirina[3], Giovanni Gamba[2], Jean François Rajaonarison[4], Gabriele Loris Beccaro[2], David Ramanitrahasimbola[2,5]

1 Laboratoire Aliments, Réactivité et Synthèse des Substances Naturelles, Faculté des Sciences et Techniques, Université des Comores, Moroni, Comoros;
2 Dipartimento di Scienze Agrarie, Forestali e Alimentari, Università degli Studi di Torino, Largo Braccini 2, Grugliasco (TO), Italy
3 Institut Malgache de Recherches Appliquées, Antananarivo, Madagascar
4 Ecole Doctorale, Génie du Vivant et Modélisation, Université de Mahajanga, Mahajanga, Madagascar
5 Pharmacy Department, Faculty of Medicine, University of Antananarivo, Antananarivo, Madagascar
* Correspondence: dario.donno@unito.it

Academic Editor: Firstname Lastname

Received: date
Revised: date
Accepted: date
Published: date

Citation: To be added by editorial staff during production.

Copyright: © 2025 by the authors. Submitted for possible open access publication under the terms and conditions of the Creative Commons Attribution (CC BY) license (https://creativecommons.org/licenses/by/4.0/).

1. Materials and Methods

1.1. Chemicals and Solvents

The following chemicals and solvents were purchased from Sigma-Aldrich (St. Louis, MO, USA): 1,1-diphenyl-2-picrylhydrazyl (DPPH), phenylephrine, acetylcholine, furosemide, sodium carbonate, Folin-Ciocalteu phenolic reagent, sodium acetate, acetic acid, citric acid, potassium chloride, hydrochloric acid, ferric chloride hexahydrate, magnesium sulfate, monopotassium phosphate, D-glucose, calcium chloride, 2,4,6-tripyridyl-S-triazine, 1,2-phenylenediamine dihydrochloride (OPDA), as well as all polyphenolic and terpenic standards, potassium dihydrogen phosphate, phosphoric acid, and HPLC grade solvents methanol and acetonitrile. Acetic acid, ethanol, organic acids, and formic acid of HPLC grade were purchased from Fluka BioChemika (Buchs, Switzerland). The disodium salt of ethylenediaminetetraacetic acid (EDTA) was obtained from AMRESCO (Solon, OH, USA). Sodium fluoride was supplied by Riedel de Haen (Seelze, Germany). Cetyltrimethylammonium bromide (cetrimide), ascorbic acid (AA), gallic acid (GA), and dehydroascorbic acid (DHAA) were purchased from Extrasynthese (Genay, France). Ultrapure Milli-Q water was produced by Sartorius Stedim Biotech, Arium model (Sartorius, Göttingen, Germany).

1.2. Standard calibration and chromatographic conditions

Quantitative analysis of different standards listed in Table 1 was determined using the external standard calibration method. Manual injection of 20 µL of each standard at the listed concentrations in Table 1 was performed in triplicate. The calibration curves were obtained by plotting the peak area (y) of the compound at each concentration level versus the sample concentration (x). High-performance liquid chromatography (HPLC) using an Agilent 1200 model equipped with a G1311A quaternary pump, a manual injection valve and a 20 µL sample loop coupled with an Agilent G1315D UV/Vis diode array detector (HPLC DAD) was used to separate the different compounds present in the prepared samples. Five distinct chromatographic methods were used for the analysis of the samples:

one for cinnamic acids and flavonoids, one for benzoic acids and catechins, one for organic acids, one for vitamins and the last one for sugars (supplementary materials). Bioactive compounds were separated in all cases through a Phenomenex Kinetex C18 column (4.6 × 150 mm, 5 µm, Agilent Technologies). All HPLC analyses were conducted in triplicate following the protocols established by **Donno *et al.* (2016)**.

1.3. Quantification of the total phenolic content (TPC)

The TPC in the leaves of *D. comorensis* was evaluated using the Folin-Ciocalteu colourimetric method described by **Slinkard and Singleton (1977)**. Leaf powders (5 g) were macerated for 24 h in the dark with 75 mL of a mixture of methanol-water (95/5; *v/v*) acidified with hydrochloric acid. The mixture was filtered through Whatman filter paper, 185 mm Ø. Filtrate (10 µL) was added to 190 µL of extraction solvent, 1 mL of Folin-Ciocalteu reagent (diluted 10 times) and 800 µL sodium carbonate (7.5%). The mixture was incubated in the dark for 30 minutes, and the absorbance was read using a UV/Vis spectrophotometer (1600-PC, VWR International) at 760 nm wavelength. Gallic acid standard solution was prepared at 0.02 - 0.10 mg/mL according to **Donno *et al.* (2016),** and the results were presented as gallic acid equivalents (GAE) per 100 g of dry weight (DW).

1.4. Antioxidant capacity

MDCR and DDCR antioxidant capacities were evaluated using both the 2,2-diphenyl-1-picrylhydrazyl (DPPH) assay described by **Sreejayan and Rao (1997)** with slight modifications and the Ferric Reducing Antioxidant Power (FRAP) assay described by **Tombozara *et al.* (2020)**.

1.4.1. DPPH free radical scavenging assay

Methanol solution MDCR and DDCR (25 µL) at different concentrations varying from 3.9 to 125 µg/mL were added to 175 µL of a DPPH solution in methanol (0.25 mmol/L) in a 96-well plate. Then, the mixtures were incubated in the dark at room temperature for 30 minutes. DPPH solution served as the negative control, while methanol was used as a blank. Gallic acid solution at different concentrations varying from 1.25 to 40 µg/mL was used as a positive control. The results were expressed as the average inhibitory concentration (IC) calculated using the following equation: **$IC (\%) = 100 \times (A_0 - A_1) / A_0$**, where A_0 and A_1 represent the absorbance values at $\lambda = 517$ nm of the negative control and the tested sample, respectively. The IC_{50} values, representing the concentration demonstrating 50% inhibition, of MDCR, DDCR and gallic acid were obtained by linear regression from three replicates.

1.4.2. Ferric Reducing Antioxidant Power (FRAP)

The ferric reducing antioxidant power (FRAP) assay relies on the reduction of Fe^{3+} to Fe^{2+} by antioxidant compounds present in *D. comorensis* leaves, measured using a 2,4,6-tripyridyl-S-triazine (TPTZ) solution (**Benzie and Strain, 1999**). Leaf powders (5 g) were macerated for 24 h in the dark with 75 mL of a mixture of methanol-water (95/5; *v/v*) acidified with 37 µL of hydrochloric acid (37%). The mixture was filtered through Whatman filter paper, 185 mm Ø. Filtrate (10 µL) was added to 20 µL of extraction solvent, 900 µL of FRAP reagent consisting of a mixture of 0.3 M acetate buffer pH 3.6, 10 mM of TPTZ in 40 mM HCl, 20 mM ferric chloride (84/8/8; *v/v/v*), 90 µL of distilled water. The final mixture was incubated at 37°C in a thermostatic bath for 30 min. Blank was prepared by replacing the sample with the extraction solvent. The absorbance was measured at a wavelength of 595 nm by using a UV/Vis spectrophotometer (model 1600 - PC, VWR International). $FeSO_4 \cdot 7H_2O$ standard solution was prepared at 100 - 1000 µmol/mL, and the results were expressed in millimoles of ferric ions equivalent (FIE) per kilogram of dried weight.

Tables

Table S1. Calibration curve, R2, LOD, and LOQ of each selected bioactive compound.

Class	Standard	Calibration curve equation	R^2	Calibration curve range (mg L^{-1})	LOD (mg L^{-1})	LOQ (mg L^{-1})
Cinnamic acids	caffeic acid	y = 59.046x + 200.6	0.996	111 - 500	0.30	1.02
	chlorogenic acid	y = 13.583x + 760.05	0.984	111 - 500	0.94	3.13
	coumaric acid	y = 8.9342x + 217.4	0.997	111 - 500	2.91	9.69
	ferulic acid	y = 3.3963x - 4.9524	1.000	111 - 500	1.24	4.15
Flavonols	hyperoside	y = 7.1322x - 4.583	0.999	111 - 500	3.37	11.24
	isoquercitrin	y = 8.3078x + 26.621	0.999	111 - 500	0.25	0.84
	quercetin	y = 3.4095x - 98.307	0.998	111 - 500	4.06	13.52
	quercitrin	y = 2.7413x + 5.6367	0.998	111 - 500	5.46	18.19
	rutin	y = 6.5808x + 30.831	0.999	111 - 500	2.94	9.79
Benzoic acids	ellagic acid	y = 29.954x + 184.52	0.998	62.5 - 250	0.61	2.04
	gallic acid	y = 44.996x + 261.86	0.999	62.5 - 250	0.44	1.45
Catechins	catechin	y = 8.9197x + 66.952	1.000	62.5 - 250	2.34	7.81
	epicatechin	y = 12.88x - 43.816	0.999	62.5 - 250	0.76	2.54
Organic acids	citric acid	y = 1.0603x - 22.092	1.000	167 - 1000	18.80	62.68
	malic acid	y = 1.415x - 80.254	0.996	167 - 1000	15.72	52.40
	oxalic acid	y = 6.4502x + 6.1503	0.998	167 - 1000	0.55	1.83
	quinic acid	y = 0.8087x - 38.021	0.998	167 - 1000	26.11	87.02
	succinic acid	y = 0.9236x - 8.0823	0.995	167 - 1000	7.14	23.78
	tartaric acid	y = 1.8427x + 15.796	1.000	167 - 1000	8.52	28.40
Vitamin C	ascorbic acid	y = 42.71x + 27.969	0.999	100 - 1000	0.84	2.79
	dehydroascorbic acid	y = 4.1628x + 140.01	0.999	30 - 300	1.09	3.65
Sugars	fructose	y = 1.8548x + 1.2324	0.9994	125 - 1000	2.325	7.751
	glucose	y = 0.1269x - 0.1107	0.9978	125 - 1000	70.228	234.092
	sucrose	y = 0.296x - 3.2202	0.9996	125 - 1000	19.308	64.359

Table S2. Chromatographic conditions of each used method.

Method	Classes of interest	Stationary phase	Mobile phase	Wavelength (nm)
A	Cinnamic acids, flavonols	KINETEX – C18 column (4.6×150 nm, 5 μm)	A: 10 mM KH_2PO_4/H_3PO_4, pH=2.8<br>B:CH_3CN	330
B	Benzoic acids, catechins	KINETEX – C18 column (4.6×150 nm, 5 μm)	A: $H_2O/CH_3OH/HCOOH$ (5:95:0.1 v/v/v), pH=2.5<br>B: $CH_3OH/HCOOH$ (100:0.1 v/v)	280
C	Organic acids	KINETEX – C18 column (4.6×150 nm, 5 μm)	A: 10 mM KH_2PO_4/H_3PO_4, pH=2.8<br>B: CH_3CN	214
D	Vitamin C	KINETEX – C18 column (4.6×150 nm, 5 μm)	A: 5 mM $C_{16}H_{33}N(CH_3)_3Br$/50 mM KH_2PO_4, pH=2.5<br>B: CH_3OH	261, 348
E	Sugars	KINETEX – C18 column (4.6×150 nm, 5 μm)	A: H_2O<br>B: CH_3CN	267, 286

Elution conditions
Method A, gradient analysis: 5%B to 21%B in 17 min + 21%B in 3 min (2 min conditioning time); flow: 1.5 mL min^{-1}
Method B, gradient analysis: 3%B to 85%B in 22 min + 85%B in 1 min (2 min conditioning time); flow: 0.6 mL min^{-1}
Method C, gradient analysis: 5%B to 14%B in 10 min + 14%B in 3 min (2 min conditioning time); flow: 0.6 mL min^{-1}
Method D, isocratic analysis: ratio of phase A and B: 95:5 in 10 min (5 min conditioning time); flow: 0.9 mL min^{-1}
Method E, isocratic analysis: ratio of phase A and B: 5:85 in 12 min (3 min conditioning time); flow: 0.5 mL min^{-1}

References

~~Benzie, I. F., Strain, J. J., 1999. Ferric reducing/antioxidant power assay: Direct measure of total antioxidant activity of biological fluids and modified version for simultaneous measurement of total antioxidant power and ascorbic acid concentration. *Methods in Enzymology*, 299, 15-27. https://doi.org/10.1016/S0076-6879(99)99005-5~~

~~Donno, D., Beccaro, G. L., Mellano, M. G., Marinoni, T. D., Cerutti, A. K., Canterino, S., Bounous, G., 2012. Application of sensory, nutraceutical and genetic techniques to create a quality profile of ancient apple cultivars. *Journal of Food Quality*, 35, 169-181. https://doi.org/10.1111/j.1745-4557.2012.00442.x~~

Donno, D., Boggia, R., Zunin, P., Cerutti, A. K., Guido, M., Mellano, M. G., Prgomet, Z., Beccaro, G. L., 2016. Phytochemical fingerprint and chemometrics for natural food preparation pattern recognition: An innovative technique in food supplement quality control. *Journal of Food Science and Technology*, *53*, 1071-1083. https://doi.org/10.1007/s13197-015-2115-6

Slinkard, K., Singleton, V. L., 1977. Total phenol analysis: automation and comparison with manual methods. *American journal of enology and viticulture*, *28*(1), 49-55. https://doi.org/10.5344/ajev.1977.28.1.49

Sreejayan N., Rao M. N. A., 1997. Nitric Oxide Scavenging by Curcuminoids. *Journal of Pharmacy and Pharmacology, 49(1)*, 105-107. https://doi.org/10.1111/j.2042-7158.1997.tb06761.x

Tombozara, N., Donno, D., Razafindrakoto, Z. R., Randriamampionona, D., Ramanitrahasimbola, D., Andrianjara, C., Ramilison-Razafimahefa, R. D., Rakotondramanana, D. A., Beccaro, G. L., 2020. The first assessment on antioxidant and antidiabetic activities of leaves and stems of *Vaccinium secundiflorum* Hook. (Ericaceae), an endemic plant of Madagascar. *South African Journal of Botany, 130*, 422-429. https://doi.org/10.1016/j.sajb.2020.01.035

Disclaimer/Publisher's Note: The statements, opinions and data contained in all publications are solely those of the individual author(s) and contributor(s) and not of MDPI and/or the editor(s). MDPI and/or the editor(s) disclaim responsibility for any injury to people or property resulting from any ideas, methods, instructions or products referred to in the content.
